# Supplementary material for: Visual Impairment Screening Assessment (VISA) tool: pilot validation
Source: BMJ Open. 2018 Mar 6;8(3):e020562. doi: 10.1136/bmjopen-2017-020562 (PMC5855179; doi:10.1136/bmjopen-2017-020562)
Supplement: Author's manuscript [file bmjopen-2017-020562.draft_revisions.pdf]

BMJ Open is committed to open peer review. As part of this commitment we make the peer review history of every article we publish publicly available.

When an article is published we post the peer reviewers' comments and the authors' responses online. We also post the versions of the paper that were used during peer review. These are the versions that the peer review comments apply to.

The versions of the paper that follow are the versions that were submitted during the peer review process. They are not the versions of record or the final published versions. They should not be cited or distributed as the published version of this manuscript.

BMJ Open is an open access journal and the full, final, typeset and author-corrected version of record of the manuscript is available on our site with no access controls, subscription charges or pay-per-view fees (http://bmjopen.bmj.com).

If you have any questions on BMJ Open's open peer review process please email info.bmjopen@bmj.com

BMJ Open

Visual Impairment Screening Assessment (VISA) tool; pilot validation.

Journal:	*BMJ Open*
Manuscript ID	bmjopen-2017-020562
Article Type:	Research
Date Submitted by the Author:	10-Nov-2017
Complete List of Authors:	Rowe, Fiona; University of Liverpool, Health Services Research<br>Hepworth, Lauren; University of Liverpool, Health Services Research<br>Hanna, Kerry; University of Liverpool, Health Services Research<br>Howard, Claire; University of Liverpool, Health Services Research
**Primary Subject Heading**:	Ophthalmology
Secondary Subject Heading:	Neurology
Keywords:	Screening, Stroke < NEUROLOGY, Visual impairment, Orthoptics, Detection

SCHOLARONE™
Manuscripts

For peer review only

Visual Impairment Screening Assessment (VISA) tool; pilot validation.

Fiona J Rowe, Lauren R Hepworth, Kerry L Hanna, Claire Howard

Department of Health Services Research, University of Liverpool, UK

Address for correspondence:

Dr Fiona Rowe

Department of Health Services Research

Waterhouse Building Block B, 2nd floor,

University of Liverpool,

1-5 Brownlow Street,

Liverpool L69 3GL

E: rowef@liverpool.ac.uk

T: 0151 7944956

Word Count: 3,280

Keywords: Screening; Stroke; Visual impairment; Orthoptics; Detection

For peer review only

For peer review only - http://bmjopen.bmj.com/site/about/guidelines.xhtml

Abstract

Objective: To report and evaluate a new Vision Impairment Screening Assessment (VISA) tool intended for use by the stroke team to improve identification of visual impairment in stroke survivors.

Design: Prospective case cohort comparative study.

Setting: Stroke units at two secondary care hospitals and one tertiary centre.

Participants: 116 stroke survivors were screened, 62 by naïve and 54 by non-naïve screeners.

Main outcome measures: Both the screening tool and comprehensive reference vision assessment measured case history, visual acuity, eye alignment, eye movements, visual field and visual inattention.

Results: Full completion of screening and reference vision assessment was achieved for 89 stroke survivors. Missing data for one or more sections typically related to patient inability to complete the assessment. Sensitivity and specificity of the screening tool was 90.24% and 85.29% respectively; the positive and negative predictive values were 93.67% and 78.36% respectively. Overall agreement was significant; K=0.736. Lowest agreement was found for screening of eye movement and visual inattention deficits.

Conclusions: Pilot validation indicates acceptability of the tool for screening of visual impairment in stroke survivors. Sensitivity and specificity were high indicating the potential accuracy of this tool for screening purposes. Results of this study have guided the revision of the VISA screening tool ahead of full clinical validation.

Strengths and limitations of this study

- Iterative development process for the screening tool.
- Prospective clinical pilot validation process.
- Comparison made between naïve and non-naïve screeners.
- Acceptability of the screening assessment to stroke survivors was not captured.
- The duration of the screening assessment was not captured.

For peer review only - http://bmjopen.bmj.com/site/about/guidelines.xhtml

BACKGROUND

Visual impairment following stroke is common and estimated to affect two thirds of all stroke survivors [1]. There is currently no standardised protocol for screening or referral and, for these patients, a considerable proportion of patients who have visual problems go unrecognised, thus receiving no advice or management [2]. There are various visual treatment options that can have a beneficial effect on vision and to general rehabilitation [3-5]. Visual impairment can have a substantial impact on quality of life including loss of confidence, impaired mobility, inability to judge distances and increased risk of falls [3]. There is a known link between poor vision, quality of life and depression in older persons [4 6]. For these reasons it is important that patients with visual impairment are identified by the stroke multidisciplinary team (MDT) and appropriate referral made for specialist vision assessment. It is equally important that the effects of visual impairment on functional ability are established and information is provided regarding the use of residual vision to facilitate general rehabilitation. These issues have been recognised as research priorities in the James Lind Alliance sight loss prioritisation process in which screening and assessment of stroke survivors for visual problems is listed as a top ten priority for research [7].

The aim of this study was to develop and evaluate a Vision Impairment Screening Assessment (VISA) tool using simple established assessments of visual function coupled with detailed instructions and tested against a reference of a full vision assessment, plus to assess the agreement of results between the screening and vision assessments.

METHODS

Development

The VISA screening tool was developed following consultation with an expert panel consisting of: stroke-specialist clinical orthoptists, stroke research orthoptists, stroke survivors with visual impairment, stroke-specialist occupational therapists and neuro-ophthalmology. The panel considered results of recent stroke/vision research studies in which multiple measures of visual function were made [2 8]. They identified the consistent vision measures across the common visual impairments occurring following stroke – those of impaired central vision, eye movement, visual field and visual attention.

Stroke survivors provided specific input on potential burden of these assessments to individuals, particularly when undertaken in the early acute stage post stroke onset. Following this panel discussion, a draft screening tool was circulated along with detailed instructions compiled for each of the screening assessments. An iterative process was followed in which the panel provided written feedback on the first and subsequent drafts of the screening tool.

For peer review only - http://bmjopen.bmj.com/site/about/guidelines.xhtml

The final pilot version of the VISA tool consists of a case history section in which visual symptoms are documented, a visual acuity section to screen central vision at near and distance, an ocular alignment and movement section to screen the presence/absence of strabismus (eye position) and eye movement problems, a visual field section to screen for peripheral field of vision, and a visual perception section to screen for visual inattention/neglect. The VISA tool provides detailed instructions regarding correct use of the assessments required for screening. This self-directed design with the incorporation of detailed instructions as part of the tool was developed on the basis that many stroke clinicians do not have any formal eye training and may not have access to such training. Thus the aim was to provide in-built instructions in lieu of formal training.

Pilot validation

A prospective case cohort comparative design was used for the pilot validation clinical study. Individuals were suitable for inclusion if they were 18 years of age or older, had clinical diagnosis of stroke as defined by World Health Organisation [9], had the ability to agree to vision screening using verbal or non-verbal indications of agreement, did not have severe cognitive impairment preventing screening and did not decline vision screening. Our inclusion criteria were intended to be pragmatic and inclusive of as many stroke survivors as possible.

Recruitment took place across three hospitals in which an orthoptist routinely screened each patient admitted to the stroke unit (as per national guidelines: Royal College of Physicians Intercollegiate Stroke Guidelines and British & Irish Orthoptic Society extended guidelines for stroke practice) to determine whether they have visual impairment [10 11]. This study collected results from these routine orthoptic vision assessments for those individuals who were also screened within 24 hours for visual impairment using the VISA screening tool. The screening tool was used by medical students and orthoptists, and always compared to a second independent vision assessment (n=5 orthoptists/ophthalmologists). Medical students (n=2) were chosen as screeners to represent completely naïve individuals in conducting vision screening assessments. Orthoptists (n=4) were also chosen as screeners in this pilot stage of validation to serve as a quality check of the screening tool's ability to accurate assess various aspects of visual impairment.

Each patient was also assessed with comprehensive vision assessment comprising: case history, visual acuity, ocular alignment and movement, visual field and visual perception. This assessment was undertaken within 24 hours (typically the same day) of the screening assessment – to minimise effect of potential recovery.

For peer review only - http://bmjopen.bmj.com/site/about/guidelines.xhtml

The order of screening and vision assessments varied to avoid the effects of fatigue and bias towards either the screen or vision assessments. The screener and orthoptist were blinded to each other's assessments to prevent bias of assessment.

Results were taken in numerical format from the referral forms completed by both the screener and orthoptist. The primary outcome measure was presence or absence of visual impairment (defined as low vision <0.2, visual field loss, eye movement abnormality, visual perceptual abnormality) and recorded as a binary measure: Yes/No for presence/absence of visual impairment.

Statistical methodology and sample size

The full vision assessment was taken as the reference standard. Kappa values assessing chance-eliminated agreement were calculated between the screening and vision assessment results. Level of sensitivity was estimated as the proportion of patients with visual impairment that are correctly identified by the screener, and the corresponding 95% confidence interval was calculated. Additionally, we estimated the level of specificity as the proportion of patients without visual impairment that are correctly identified by the screener, and the corresponding 95% confidence interval. Further, we calculated the positive and negative predictive values for screening assessment. As this was a pilot validation study, we sought to include a minimum sample size of 100 subjects. This sample size is typically used for diagnostic accuracy studies, which we considered appropriate even though this was a study of screening detection rather than diagnostic accuracy [12].

Process evaluation

Process evaluation for acceptability of the VISA tool was through a combination of feedback sheets and one-to-one interviews with screeners. Interviews and feedback sheets were transcribed and all identifying features removed. Qualitative data analysis was undertaken as an on-going iterative process. All transcripts were systematically coded manually. A thematic approach to analysis of the qualitative data was adopted. Codes were grouped for similar content and these groups defined the key emerging themes. A modified grounded theory approach was undertaken in which themes were revised iteratively as further interviews and analysis progressed.

RESULTS

Completion rate

One hundred and sixteen stroke patients (67% female, mean age 68.9 years) received both a VISA screening assessment and a reference vision assessment, over a four-month period. Two medical students conducted 62 of the screening assessments and 54 were screened by a team of four

For peer review only - http://bmjopen.bmj.com/site/about/guidelines.xhtml

orthoptists. Independent full vision assessment was conducted by a team of four orthoptists and one ophthalmologist.

The screening assessment was fully completed by 89 patients, with the remaining 28 missing one or more elements (n=4 near vision, n=6 distance vision, n=3 convergence, n=9 visual fields, n=28 visual inattention). The vision assessment was fully completed by 77 patients, with the remaining 40 missing one or more elements (n=3 near vision, n=9 distance vision, n=18 convergence, n=9 visual fields, n=23 visual inattention). Reasons for missing data were captured and typically related to patient inability to complete sections of vision assessments because of impaired cognitive ability or fatigue.

For peer review only

Referral agreement

The agreement of whether to make a referral to specialist eye services based on the results of the screening tool versus those from full vision assessment had a Kappa value of 0.736 (95% CI 0.602 – 0.870).

In this pilot evaluation of the VISA screening tool, sensitivity of 90.24% and specificity of 85.29% were found. The positive and negative predictive values were 93.67% and 78.36% respectively. These calculations are outlined in Table 1.

Table 1: Calculations of sensitivity, specificity and predictive values

Positive	
True positive	74
False negative	8
Negative	
False positive	5
True negative	29
Output	
Sensitivity (true positive/true positive + false negative)	90.24% (95% CI: 81.68 – 95.69%)
Specificity (true negative/false positive + true negative)	85.29% (95% CI: 68.94 – 95.05%)
Positive predictive value (true positive/false positive + true positive)	93.67% (95% CI: 86.78 – 97.09%)
Negative predictive value (true negative/false negative + true negative)	78.38% (95% CI: 64.91 – 87.66%)

For peer review only - http://bmjopen.bmj.com/site/about/guidelines.xhtml

Agreement was found for 103 participants (29 had no visual impairment, 74 required referral because of failed screening), outlined in Figure 1. The screening assessment produced eight false negative and five false positive results. Of the false negative results, three had ocular motility problems, three had reduced distance vision, one had reduced near vision and one did not have visual fields tested during screening. For false positive results, two with visual inattention, two with visual field loss and one with both visual inattention and visual field loss, were detected by screening and found not to be present by the vision assessment.

Test component agreement

The agreement for the individual components between the screening tool and vision assessments are outlined in Table 2. The highest levels of agreement were produced for distance visual acuity (0.785) and visual fields (0.741). The lowest levels of agreement were produced for ocular motility (0.120) and visual inattention (0.361). Low agreement for ocular motility related to high false negatives where 21 cases (3 with multiple conditions) were not detected - these comprised of: nine defects of vertical movement (including four age-related restrictions, one 4th cranial nerve palsy and one V-pattern), eight cases of nystagmus (including four end-point nystagmus), five restrictions of horizontal eye movements and four cases of reduced convergence. The low agreement with visual inattention related to false positive referrals because of failure of the patient to complete this section due to impaired cognitive ability or fatigue – rather than true presence of visual inattention.

Table 2: Summary of agreement between screening tool and vision assessment for referral to specialist eye services and individual components

Element of testing	Agreement	False negative	False positive	Kappa value (95% CI)
Referral	103	8	5	0.736 (0.602 – 0.870)
Near visual acuity	93	10	7	0.682 (0.543 – 0.820)
Distance visual acuity	94	8	3	0.785 (0.665 – 0.904)
Ocular alignment	112	4	0	0.585 (0.221 – 0.949)
Ocular motility	89	21	6	0.120 (-0.071 – 0.311)
Visual fields	94	3	8	0.741 (0.599 – 0.884)
Visual inattention	67	1	16	0.361 (0.144 – 0.578)

For peer review only - http://bmjopen.bmj.com/site/about/guidelines.xhtml

Naïve versus non-naïve screeners

The agreement on whether to make a referral to specialist eye services based on results of the screening tool versus those from full vision assessment was stronger when made by a non-naïve screener (Table 3). A higher rate of false positive and false negatives were found when the screener was naïve to vision testing (eleven false referrals for naïve vs two for non-naïve screeners). The agreement on whether to make a referral to specialist eye services between the screening tool and a vision assessment had a Kappa value of 0.736 (95% CI 0.602 – 0.870).

When used by a naïve screener the VISA tool has a sensitivity of 82.93% and specificity of 80.95%. When used by non-naïve screeners the sensitivity the tool has a sensitivity of 97.56% and specificity of 92.31%.

Table 3: Summary of agreement between screening tool and vision assessment for referral to specialist eye services when used by a naïve versus non-naïve screener.

Screener	Agreement	False negative	False positive	Kappa value (95% CI)
Medical student<br>n=62	51	7	4	0.617 (0.415 – 0.820)
Independent orthoptist<br>n=54	52	1	1	0.899 (0.761 – 1.000)

Process evaluation

Information from feedback sheets and detailed notes from interviews were compiled and grouped for type of feedback. Group themes included instruction feedback, section feedback and referral feedback.

Instruction feedback: Screeners asked for brief instruction reminders at the top of screening assessments, for example, position test chart at 3 metres from the patient, cover each eye in turn, etc. This served to act as a quick reminder for the correct procedure for that particular section of the screening tool. Clarifications were requested for the main instruction training section such that potential ambiguity was removed.

Section feedback: In the first version, each screening section was coupled to the detailed assessment instructions. Screeners requested that all detailed instructions be merged into one training 'manual' section with the screening assessments separate. As screeners became more familiar with the tool, they used the screening assessments on their own and kept the detailed instructions elsewhere (mainly for reference) which meant there was less paperwork to be carried to the bedside assessment.

For peer review only - http://bmjopen.bmj.com/site/about/guidelines.xhtml

Referral feedback: Most feedback concerned patients who were borderline on whether to refer for specialist vision assessment or not. For example, where the patient had borderline visual acuity responses – perhaps because glasses were not available – but all other visual function assessments passed the screening assessment. In other cases, the patient lacked sufficient cognitive or communication abilities rendering some screening assessments 'unsure' or incomplete. Detailed referral guidelines were compiled to guide the referral process with minimum guidance being to repeat the screening assessment 1-2 days later for borderline cases. This aimed to reduce the levels of false referrals.

For peer review only

DISCUSSION

In this study, we present the VISA screening tool which encompasses screening of key visual functions affected by stroke; namely central vision, peripheral visual field, eye position/movements and visual attention, alongside ocular history. Overall, referral had sensitivity and specificity of about 90% and 80% respectively, positive and genitive predictive values of about 94% and 78% respectively, with agreement between screening and comprehensive assessment of above Kappa 0.7. Agreement was lowest for eye movement screening and visual inattention whereas all other individual sections showed agreement of above Kappa 0.5. Low agreement in these sections related to high false positive referrals where screening assessment indicated a fail for ocular motility or visual inattention. The full vision assessment detected ocular motility changes but these would be classed as 'normal' physiological eye movement patterns such as V pattern and end-point nystagmus, which alone would not have instigated referral. The detection of these physiological eye movement patterns was regarded as a negative finding within the eye movement section indicating that the ocular motility section had proved to be sensitive to these less obvious eye movement problems. However, this section requires close monitoring in further studies to refine related training and referral guidelines. False positive referrals for visual inattention occurred where the patient failed to complete the section because of fatigue or cognitive impairment. The incomplete results were interpreted as borderline fail by screeners. Visual inattention was the last section to be completed in the screening assessment so, as a result, likely to be most susceptible to the effects of fatigue and impaired cognition. Guidance on completing the screening assessment was therefore amended such that the more interactive components of the screening assessment were advised to be completed first in such cases plus a repeat second screen where indicated.

Process evaluation aided further refinement of the screening tool and, in particular, training elements and referral guidance to add quick tips and reminders, and to remove ambiguity. Vision screening of stroke survivors by orthoptists using validated assessments has been shown to provide

For peer review only - http://bmjopen.bmj.com/site/about/guidelines.xhtml

accurate identification of visual impairment and is easily undertaken on the stroke unit with further follow-up arranged in eye clinics as required [13]. Such Orthoptic input has been reported to help prevent misdiagnosis, provide quick access to treatment of visual problems and improve response to general rehabilitation [4 14]. Orthoptists are a member of the core acute stroke MDT (10). Despite consistent findings that inclusion of vision services within the MDT is highly beneficial, such visual assessment is not common and services are inconsistent throughout the UK. One survey showed that 45% of stroke services provided no formal vision assessment for stroke patients [15]. A further survey of practice identified that only 7% of stroke units had a policy relating to vision assessment and management [16]. Both surveys showed lack of standardisation for vision assessment and treatment for stroke survivors. The National Stroke Strategy argues that vision and visual perceptual difficulties are components requiring multi-faceted stroke specific rehabilitation and support [17]. The Royal College of Physicians recommend that every patient with stroke should have a practical assessment of vision and examination of the visual field [10].

Problems exist with referral accuracy from the MDT where there is suspected visual difficulty. It is reported that where referral by the MDT was based on the identification of ocular signs *only*, there was reduced sensitivity (42%) and specificity (52%). Referral accuracy improved when visual symptoms were taken into account. Concerns were raised regarding potential failure to refer those patients unable to report their visual symptoms due to communication and cognitive deficits [3]. Inconsistencies between identification of ocular signs on assessment by the MDT and final ocular diagnosis have also been documented in an audit of stroke referrals for vision assessment. Fifty-six percent of visual diagnoses made prior to formal eye assessment were incorrect with amended diagnoses being made following visual assessment by the orthoptic/ophthalmic team [18]. Our VISA screen at this early pilot stage appears to increase the accuracy of screening by increasing the ability and detect ocular signs separate to reporting of vision symptoms.

In each of the above studies, the MDT used a screening form on which they specified whether they noted any obvious visual signs such as nystagmus, strabismus or ptosis and whether the patient complained of visual symptoms such as double vision or reading difficulty. They did not, however, undertake any measurement of visual function. A further study evaluated Cardiff cards as a screening measure to identify low levels of vision [19]. A comparative study of qualitative methods of visual field assessment reported the difficulty in screening for visual field impairment in acute stages of stroke follow-up [20]. However, the authors recognised that confrontation is widely regarded as the most viable screening option for bedside visual field assessment (19). Visual inattention is the most common visual perceptual disorder and there are various screening assessments in use for its

For peer review only - http://bmjopen.bmj.com/site/about/guidelines.xhtml

detection but which do not extend to other facets of visual impairment [21]. In each of these studies, individual assessments of one aspect of visual function are considered. However, an overall visual screening assessment for stroke survivors is currently not available for use by MDTs in the absence of assessment by eye care professionals [21].

Limitations: The VISA screening tool was used by a combination of medial students and orthoptists whilst full vision assessment was provided by a team of orthoptists and ophthalmologists. Arguably, results wold be more meaningful if all screening assessments were completed by staff naïve to any vision assessment. Because this was a pilot validation study, we chose to include screening assessments from both medical students with no vision assessment experience, and orthoptists who were experienced in vision assessment. Medical students represented completely naïve individuals in conducting vision screening assessments. However, orthoptists were chosen as screeners in this pilot stage of validation to serve as a quality check of the screening tool's ability to accurate assess various aspects of visual impairment. Our process evaluation for acceptability of the screening assessment involved feedback and interviewers with screeners only. We acknowledge this limitation and an important next step is to obtain views of stroke survivors on the acceptability of the screening assessment and its perceived value to them. A further limitation is that the screening assessment was not timed consistently for duration. Completion of the screening assessment was approximately 10 minutes in the small number that could be assessed but this cannot be taken as a representative screen duration. The screening duration is an important consideration when adding to busy acute stroke services and will be captured fully in the next stage of validation.

Our next stage of development is a full clinical validation of the VISA tool where all screening assessments are completed by naïve screeners versus reference comprehensive vision assessment.

CONCLUSIONS

This early validation of the VISA screening tool shows promise in improving detection accuracy with potential to lead to more prompt referral with fewer false positives and negatives. The benefits are that it may support increased speed of access to appropriate treatment of visual impairment and potential to preserve and make best use of remaining visual function for patients. Identification of visual impairment and implementation of early interventions and compensatory options has impact to overall rehabilitation, quality of life and activities of daily living with potential cost savings to the NHS by enhancing rehabilitation and supporting early discharge. Establishment of an effective vision screening tool is likely to be highly transferable to other vulnerable groups in other hospital in-patient areas, residential care settings or community multidisciplinary team assessments.

For peer review only - http://bmjopen.bmj.com/site/about/guidelines.xhtml

Funding statement

This study received no specific grant from any funding agency in the public, commercial or not-for-profit sectors'. FR and CH are part funded by National Institute for Health Research fellowship awards.

Competing interests

The authors have no competing interests to declare.

Acknowledgements

The authors would like to express thanks to the staff and patients at Salford Royal Hospital and the Walton Centre.

Author contributions

FR designed and wrote the study protocol. All authors (FR, LH, KH and CH) were involved in study set-up and data collection. LH and FR carried out the data analysis and wrote the initial manuscript draft. The manuscript was critically reviewed by KH and CH. All authors read and approved the final manuscript.

Data sharing

No additional data available

REFERENCES

1. Hepworth LR, Rowe FJ, Walker MF, et al. Post-stroke visual impairment: a systematic literature review of types and recovery of visual conditions. *Ophthalmology Research* 2016;5(1) doi: 10.9734/OR/2016/21767
2. Rowe F, VIS writing group. Vision In Stroke cohort: profile overview of visual impairment. *Brain and Behavior* 2017 doi: 10.1002/brb3.771
3. Rowe F, Wright D, Brand D, et al. Reading difficulty after stroke: ocular and non ocular causes. *International Journal of Stroke* 2011;6(5):404-11.
4. Jones SA, Shinton RA. Improving outcome in stroke patients with visual problems. *Age and Ageing* 2006;35(6):560-65.
5. Hanna KL, Hepworth LR, Rowe FJ. The treatment methods for post-stroke visual impairment: a systematic review. *Brain and Behavior* 2017;7(5) doi: 10.1002/brb3.682
6. Tsai S-Y, Cheng C-Y, Hsu W-M, et al. Association between visual impairment and depression in the elderly. *Journal of Formosan Medical Association* 2003;102(2):86-90.
7. The James Lind Alliance. Top 10s of priorities for research 2017 [Available from: http://www.jla.nihr.ac.uk/top-10-priorities/ accessed 13 June 2017.
8. Rowe F. Impact of Visual Impairment after Stroke: the IVIS study 2014 [Available from: www.ivis.org.uk/.

For peer review only - http://bmjopen.bmj.com/site/about/guidelines.xhtml

9. Aho K, Harmsen P, Hatano S, et al. Cerebrovasular disease in the community: results of a WHO Collaborative Study. *Bulletin of the World Health Organisation* 1980;58(1):113-30.
10. Intercollegiate Stroke Working Party. National clinical guideline for stroke. 5th ed. London: Royal College of Physicians 2016.
11. British and Irish Orthoptic Society. Compentency standards and professional practice guidelines: extended practioner role Section 2: Stroke assessment and rehabilitation. London: British and Irish Orthoptic Society, 2011.
12. Bland JM, Altman DG. Statistical methods for assessing agreement between two methods of clinical measurement. *Lancet* 1986;327(8476):307-10.
13. MacIntosh C. Stroke re-visited: visual problems following stroke and their effect on rehabilitation. *British Orthoptic Journal* 2003;60:10-14.
14. Rowe F, Brand D, Jackson CA, et al. Visual impairment following stroke: do stroke patients require vision assessment? *Age and Ageing* 2009;38(2):188-93.
15. Rowe FJ. Who sees visual impairment following stroke? *Strabismus* 2010;18(2):37-40.
16. Pollock A, Brady M, Hazelton C. Management of visual problems after stroke: a survey of current practice in Scotland. *International Journal of Stroke* 2009;4(Suppl 2):44.
17. Department of Health. National Stroke Strategy. London: DH Publications, 2007.
18. Brand D. Paralell Vision: British and Irish Orthoptic Society, 2009:1-2.
19. Johansen A, White S, Waraisch P. Screening for visual impairment in older people: validation of the Cardiff Acuity Test. *36* 2003;3(289-293)
20. Cassidy TP, Bruce DW, Gray CS. Visual field loss after stroke: confrontation and perimetry in the assessment of recovery. *Journal of Stroke and Cerebrovascular Diseases* 2001;10(3):113-17.
21. Hanna KL, Hepworth LR, Rowe FJ. Screening methods for post-stroke visual impairment: a systematic review. *Disability and Rehabilitation* 2017;39(25):2531-43.

For peer review only

For peer review only - http://bmjopen.bmj.com/site/about/guidelines.xhtml

Figure 1: Flow diagram of participant outcome for screening and full assessment

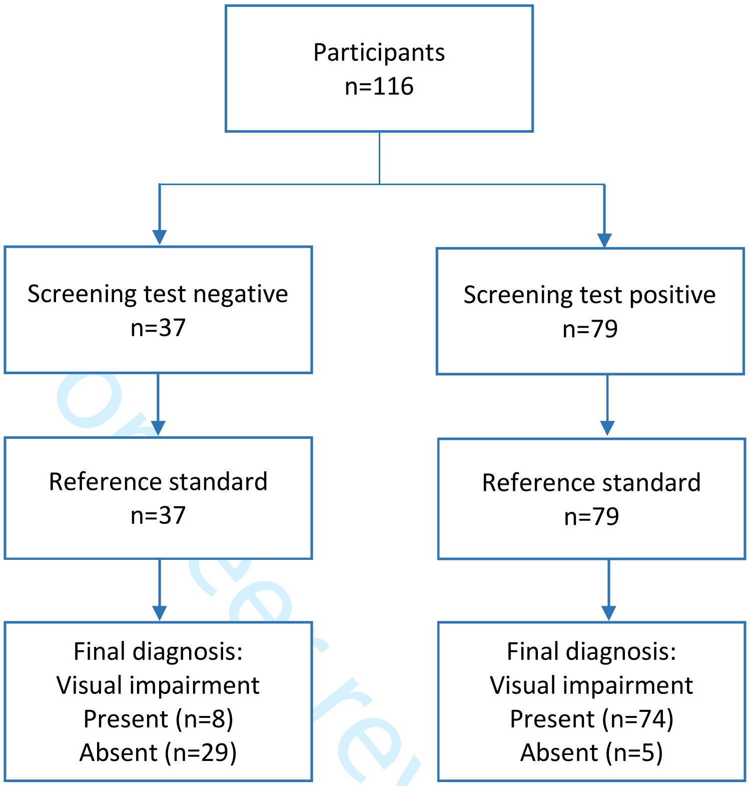

Section & Topic	No	Item	Reported on page #
**TITLE OR ABSTRACT**			
	**1**	Identification as a study of diagnostic accuracy using at least one measure of accuracy (such as sensitivity, specificity, predictive values, or AUC)	#2
**ABSTRACT**			
	**2**	Structured summary of study design, methods, results, and conclusions (for specific guidance, see STARD for Abstracts)	#2
**INTRODUCTION**			
	**3**	Scientific and clinical background, including the intended use and clinical role of the index test	#3
	**4**	Study objectives and hypotheses	#3
**METHODS**			
Study design	**5**	Whether data collection was planned before the index test and reference standard were performed (prospective study) or after (retrospective study)	#4
Participants	**6**	Eligibility criteria	#4
	**7**	On what basis potentially eligible participants were identified (such as symptoms, results from previous tests, inclusion in registry)	#4
	**8**	Where and when potentially eligible participants were identified (setting, location and dates)	#4
	**9**	Whether participants formed a consecutive, random or convenience series	#4
Test methods	**10a**	Index test, in sufficient detail to allow replication	#4
	**10b**	Reference standard, in sufficient detail to allow replication	#4
	**11**	Rationale for choosing the reference standard (if alternatives exist)	#4
	**12a**	Definition of and rationale for test positivity cut-offs or result categories of the index test, distinguishing pre-specified from exploratory	#5
	**12b**	Definition of and rationale for test positivity cut-offs or result categories of the reference standard, distinguishing pre-specified from exploratory	#5
	**13a**	Whether clinical information and reference standard results were available to the performers/readers of the index test	#5
	**13b**	Whether clinical information and index test results were available to the assessors of the reference standard	#5
Analysis	**14**	Methods for estimating or comparing measures of diagnostic accuracy	#5
	**15**	How indeterminate index test or reference standard results were handled	#5
	**16**	How missing data on the index test and reference standard were handled	#6
	**17**	Any analyses of variability in diagnostic accuracy, distinguishing pre-specified from exploratory	#7
	**18**	Intended sample size and how it was determined	#5
**RESULTS**			
Participants	**19**	Flow of participants, using a diagram	Figure 1
	**20**	Baseline demographic and clinical characteristics of participants	#5
	**21a**	Distribution of severity of disease in those with the target condition	
	**21b**	Distribution of alternative diagnoses in those without the target condition	
	**22**	Time interval and any clinical interventions between index test and reference standard	#4
Test results	**23**	Cross tabulation of the index test results (or their distribution) by the results of the reference standard	#6-7
	**24**	Estimates of diagnostic accuracy and their precision (such as 95% confidence intervals)	#6
	**25**	Any adverse events from performing the index test or the reference standard	
**DISCUSSION**			
	**26**	Study limitations, including sources of potential bias, statistical uncertainty, and generalisability	#11
	**27**	Implications for practice, including the intended use and clinical role of the index test	#11
**OTHER INFORMATION**			
	**28**	Registration number and name of registry	
	**29**	Where the full study protocol can be accessed	
	**30**	Sources of funding and other support; role of funders	#12

For peer review only

STARD 2015

AIM

STARD stands for “Standards for Reporting Diagnostic accuracy studies”. This list of items was developed to contribute to the completeness and transparency of reporting of diagnostic accuracy studies. Authors can use the list to write informative study reports. Editors and peer-reviewers can use it to evaluate whether the information has been included in manuscripts submitted for publication.

EXPLANATION

A **diagnostic accuracy study** evaluates the ability of one or more medical tests to correctly classify study participants as having a **target condition.** This can be a disease, a disease stage, response or benefit from therapy, or an event or condition in the future. A medical test can be an imaging procedure, a laboratory test, elements from history and physical examination, a combination of these, or any other method for collecting information about the current health status of a patient.

The test whose accuracy is evaluated is called **index test.** A study can evaluate the accuracy of one or more index tests. Evaluating the ability of a medical test to correctly classify patients is typically done by comparing the distribution of the index test results with those of the **reference standard**. The reference standard is the best available method for establishing the presence or absence of the target condition. An accuracy study can rely on one or more reference standards.

If test results are categorized as either positive or negative, the cross tabulation of the index test results against those of the reference standard can be used to estimate the **sensitivity** of the index test (the proportion of participants *with* the target condition who have a positive index test), and its **specificity** (the proportion *without* the target condition who have a negative index test). From this cross tabulation (sometimes referred to as the contingency or “2x2” table), several other accuracy statistics can be estimated, such as the positive and negative **predictive values** of the test. Confidence intervals around estimates of accuracy can then be calculated to quantify the statistical **precision** of the measurements.

If the index test results can take more than two values, categorization of test results as positive or negative requires a **test positivity cut-off**. When multiple such cut-offs can be defined, authors can report a receiver operating characteristic (ROC) curve which graphically represents the combination of sensitivity and specificity for each possible test positivity cut-off. The **area under the ROC curve** informs in a single numerical value about the overall diagnostic accuracy of the index test.

The **intended use** of a medical test can be diagnosis, screening, staging, monitoring, surveillance, prediction or prognosis. The **clinical role** of a test explains its position relative to existing tests in the clinical pathway. A replacement test, for example, replaces an existing test. A triage test is used before an existing test; an add-on test is used after an existing test.

Besides diagnostic accuracy, several other outcomes and statistics may be relevant in the evaluation of medical tests. Medical tests can also be used to classify patients for purposes other than diagnosis, such as staging or prognosis. The STARD list was not explicitly developed for these other outcomes, statistics, and study types, although most STARD items would still apply.

DEVELOPMENT

This STARD list was released in 2015. The 30 items were identified by an international expert group of methodologists, researchers, and editors. The guiding principle in the development of STARD was to select items that, when reported, would help readers to judge the potential for bias in the study, to appraise the applicability of the study findings and the validity of conclusions and recommendations. The list represents an update of the first version, which was published in 2003.

More information can be found on http://www.equator-network.org/reporting-guidelines/stard.

cc BY SA

BMJ Open

Visual Impairment Screening Assessment (VISA) tool; pilot validation.

Journal:	*BMJ Open*
Manuscript ID	bmjopen-2017-020562.R1
Article Type:	Research
Date Submitted by the Author:	15-Jan-2018
Complete List of Authors:	Rowe, Fiona; University of Liverpool, Health Services Research<br>Hepworth, Lauren; University of Liverpool, Health Services Research<br>Hanna, Kerry; University of Liverpool, Health Services Research<br>Howard, Claire; University of Liverpool, Health Services Research
**Primary Subject Heading**:	Ophthalmology
Secondary Subject Heading:	Neurology
Keywords:	Screening, Stroke < NEUROLOGY, Visual impairment, Orthoptics, Detection

SCHOLARONE™
Manuscripts

For peer review only

Visual Impairment Screening Assessment (VISA) tool; pilot validation.

Fiona J Rowe, Lauren R Hepworth, Kerry L Hanna, Claire Howard

Department of Health Services Research, University of Liverpool, UK

Address for correspondence:

Prof Fiona Rowe

Department of Health Services Research

Waterhouse Building Block B, 2nd floor,

University of Liverpool,

1-5 Brownlow Street,

Liverpool L69 3GL

E: rowef@liverpool.ac.uk

T: 0151 7944956

Word Count: 4,087

Keywords: Screening; Stroke; Visual impairment; Orthoptics; Detection

For peer review only

For peer review only - http://bmjopen.bmj.com/site/about/guidelines.xhtml

Abstract

Objective: To report and evaluate a new Vision Impairment Screening Assessment (VISA) tool intended for use by the stroke team to improve identification of visual impairment in stroke survivors.

Design: Prospective case cohort comparative study.

Setting: Stroke units at two secondary care hospitals and one tertiary centre.

Participants: 116 stroke survivors were screened, 62 by naïve and 54 by non-naïve screeners.

Main outcome measures: Both the VISA screening tool and comprehensive specialist vision assessment measured case history, visual acuity, eye alignment, eye movements, visual field and visual inattention.

Results: Full completion of VISA tool and specialist vision assessment was achieved for 89 stroke survivors. Missing data for one or more sections typically related to patient inability to complete the assessment. Sensitivity and specificity of the VISA screening tool was 90.24% and 85.29% respectively; the positive and negative predictive values were 93.67% and 78.36% respectively. Overall agreement was significant; K=0.736. Lowest agreement was found for screening of eye movement and visual inattention deficits.

Conclusions: This early validation of the VISA screening tool shows promise in improving detection accuracy for clinicians involved in stroke care who are not specialists in vision problems and lack formal eye training, with potential to lead to more prompt referral with fewer false positives and negatives. Pilot validation indicates acceptability of the VISA tool for screening of visual impairment in stroke survivors. Sensitivity and specificity were high indicating the potential accuracy of the VISA tool for screening purposes. Results of this study have guided the revision of the VISA screening tool ahead of full clinical validation.

For peer review only

Strengths and limitations of this study

- Iterative development process for the screening tool.
- Prospective clinical pilot validation process.
- Comparison made between naïve and non-naïve screeners.
- Acceptability of the screening assessment to stroke survivors was not captured.
- The duration of the screening assessment was not captured.

For peer review only - http://bmjopen.bmj.com/site/about/guidelines.xhtml

Background

Visual impairment following stroke is common and estimated to affect two thirds of all stroke survivors (1). There is currently no standardised protocol for screening or referral and, as a result of poor/absent screening, a considerable proportion of patients who have visual problems go unrecognised, thus receiving no advice or management (2). There are various visual treatment options that can have a beneficial effect on vision and to general rehabilitation (3-5). Visual impairment can have a substantial impact on quality of life including loss of confidence, impaired mobility, inability to judge distances and increased risk of falls (3). There is a known link between poor vision, quality of life and depression in older persons (4, 6). For these reasons it is important that patients with visual impairment are identified by the stroke multidisciplinary team (MDT) and appropriate referral made for specialist vision assessment. It is equally important that the effects of visual impairment on functional ability are established and information is provided regarding the use of residual vision to facilitate general rehabilitation. These issues have been recognised as research priorities in the James Lind Alliance sight loss prioritisation process, in which screening and assessment of stroke survivors for visual problems is listed as a top ten priority for research (7).

The overall aim of this study was to develop and evaluate a Vision Impairment Screening Assessment (VISA) tool using simple established assessments of visual function coupled with detailed instructions. Our objectives were to test the VISA screen against a reference of a specialist vision assessment to determine sensitivity, specificity, predictive values and inter-rater agreement of results between the VISA screen and specialist vision assessments. A final objective was to evaluate user views on the acceptability of use of the VISA screening tool.

Methods

Development

The VISA screening tool was developed following consultation with an expert panel consisting of: stroke-specialist clinical orthoptists, stroke research orthoptists, stroke survivors with visual impairment, stroke-specialist occupational therapists and neuro-ophthalmologists. The panel considered results of recent stroke/vision research studies in which multiple measures of visual function were made (2, 8). They identified the consistent vision measures across the common visual impairments occurring following stroke – those of impaired central vision, eye movement, visual field and visual inattention (the vision modality of spatial neglect).

Stroke survivors provided specific input on potential burden of these assessments to individuals, particularly when undertaken in the early acute stage post stroke onset. Following this panel discussion, a draft screening tool was circulated along with detailed instructions compiled for each of

For peer review only - http://bmjopen.bmj.com/site/about/guidelines.xhtml

the screening assessments, which comprised a screen of visual symptoms and observed signs, visual acuity, eye alignment and movements, visual field boundaries and visual inattention. An iterative process was followed in which the panel provided written feedback on the first and subsequent drafts of the screening tool. Feedback from both clinicians and stroke survivors guided the revision of the symptom history section to reduce the number of questions being asked and refine the question wording to remove potential ambiguity. Feedback specifically from clinicians also guided the revision of the self-guided instructions to provide more steps and detail plus to remove potential ambiguity.

The final pilot version of the VISA tool contained the same five sections as the original draft, consisting of a case history section in which visual symptoms and observed signs are documented, a visual acuity section to screen central vision at near and distance using logMAR and N-series letters, an ocular alignment and movement section to screen the presence/absence of strabismus (eye position) and eye movement problems, a visual field section to screen for peripheral field of vision by a guided confrontation method, and a visual perception section to screen for visual inattention/neglect using a triad of line bisection, cancellation task and clock drawing assessments. The VISA tool provides detailed instructions regarding correct use of the assessments required for screening. This self-directed design with the incorporation of detailed instructions as part of the tool was developed on the basis that many stroke clinicians do not have any formal eye training and may not have access to such training. Thus the aim was to provide in-built instructions in lieu of formal training.

Pilot validation

A prospective case cohort comparative design was used for the pilot validation clinical study. Individuals were suitable for inclusion if they were 18 years of age or older, had clinical diagnosis of stroke as defined by World Health Organisation (9), had the ability to agree to vision screening using verbal or non-verbal indications of agreement, did not have severe cognitive impairment preventing screening and did not decline vision screening. Our inclusion criteria were intended to be pragmatic and inclusive of as many stroke survivors as possible. The clinical study was undertaken in accordance with the Tenets of Helsinki with NHS research ethical approval.

For the purpose of this study, vision screening was undertaken with the VISA screening tool and screening was defined as the assessment of stroke survivors for the presence of reduced visual function against pre-set abnormality criteria. Specialist visual assessment was defined as the vision assessment undertaken by eye-trained clinicians (orthoptists and ophthalmologists) in which

For peer review only - http://bmjopen.bmj.com/site/about/guidelines.xhtml

detection of visual impairment was coupled with formal diagnosis of the type of visual condition present.

Recruitment took place across three hospitals in which an orthoptist was a member of the core acute stroke unit multidisciplinary team (as per national guidelines: Royal College of Physicians Intercollegiate Stroke Guidelines and British & Irish Orthoptic Society extended guidelines for stroke practice) (10, 11).

Each stroke survivor underwent two vision assessments: the routine orthoptic specialist vision assessment (n=5 orthoptists/ophthalmologists) and the VISA screening assessment. The VISA screen was completed by medical students and orthoptists. Medical students (n=2) were chosen as screeners to represent completely naïve individuals in conducting vision screening assessments. Orthoptists (n=4) were also chosen as screeners in this pilot stage of validation to serve as a quality check of the screening tool's ability to accurate assess various aspects of visual impairment.

.

Routine specialist vision assessment comprised detailed diagnostic assessments of case history, visual acuity, ocular alignment and movement, visual field and visual perception. This assessment was undertaken within 24 hours (typically the same day) of the VISA screen – to minimise effect of potential recovery.

The order of the VISA screening and specialist vision assessments varied to avoid the effects of fatigue and bias towards either the screen or vision assessments. The screener and orthoptist were blinded to each other's assessments to prevent bias of assessment. The within-assessment order of testing varied for the specialist assessment. However, the order of testing within the VISA screen followed a set order of 1) case history, 2) visual acuity, 3) eye position, 4) visual field and 5) visual inattention assessments.

Statistical methodology and sample size

Results were taken in numerical format from the referral forms completed by both the screener and orthoptist. The specialist vision assessment was taken as the reference standard.

The primary outcome measure was presence or absence of visual impairment (defined as low vision <0.2, visual field loss, eye movement abnormality, visual perceptual abnormality) and recorded as a binary measure: Yes/No for presence/absence of visual impairment. The primary outcome measure was evaluated by Kappa values assessing chance-eliminated agreement between the VISA screening and specialist vision assessment results.

For peer review only - http://bmjopen.bmj.com/site/about/guidelines.xhtml

Secondary outcome measures were the calculation of sensitivity, specificity and predictive values. Level of sensitivity was estimated as the proportion of patients with visual impairment that are correctly identified by the screener, and the corresponding 95% confidence interval was calculated. Level of specificity was estimated as the proportion of patients without visual impairment that are correctly identified by the screener, and the corresponding 95% confidence interval. Further, we calculated the positive and negative predictive values for the VISA screen.

As this was a pilot validation study, we sought to include a minimum sample size of 100 subjects. This sample size is typically used for diagnostic accuracy studies, which we considered appropriate even though this was a study of screening detection rather than diagnostic accuracy (12).

Process evaluation

Process evaluation for acceptability of the VISA tool during the clinical study was through a combination of feedback sheets and one-to-one interviews with screeners. Feedback sheets could be returned at any time during the study to report any issues with testing alongside obtaining clinician views based on their use of the VISA tool. Feedback sheets asked the following:

1. Are the instructions for the various tests clear?
2. Which instructions should be amended?
3. What additional instruction information/rewording do you suggest?
4. Which instructions require less information?
5. Are any tests not useful or difficulty to do? (Specify)
6. Should any other tests be added in?
7. How long does it take you to do the screen?
8. Other comments?

These questions were also asked during individual interviews. Interviews were conducted by the lead author (FR).

Interviews and feedback sheets were transcribed verbatim and all identifying features removed. Qualitative data analysis was undertaken as an on-going iterative process. All transcripts were systematically coded manually. A thematic approach to analysis of the qualitative data was adopted. Transcripts were coded by sentence or section and the code descriptors were derived directly from the text. A thematic approach to analysis of the qualitative data was adopted. Codes were grouped for similar content and these groups defined the key emerging themes. A modified grounded theory approach was undertaken in which themes were revised iteratively as further interviews and analysis progressed.

For peer review only - http://bmjopen.bmj.com/site/about/guidelines.xhtml

Results

Completion rate

One hundred and sixteen stroke patients received both a VISA screening assessment and a reference vision assessment, over four months (Dec 2015-Mar 2016). Two medical students conducted 62 of the VISA screens and 54 were screened by a team of four orthoptists. Independent specialist vision assessment was conducted by a team of four orthoptists and one ophthalmologist.

The VISA screen was fully completed by 89 patients, with the remaining 28 missing one or more elements (n=4 near vision, n=6 distance vision, n=3 convergence, n=9 visual fields, n=28 visual inattention). The specialist vision assessment was fully completed by 77 patients, with the remaining 40 missing one or more elements (n=3 near vision, n=9 distance vision, n=18 convergence, n=9 visual fields, n=23 visual inattention). Reasons for missing data were captured and typically related to patient inability to complete sections of vision assessments because of impaired cognitive ability or fatigue. All patients were included even if there was missing data – missing data did not automatically result in failure for that section, thereby requiring referral. Reasons behind the failure to complete sections were always taken into consideration.

Referral agreement

The agreement of whether to make a referral to specialist eye services based on the results of the VISA screening tool versus those from specialist vision assessment had a Kappa value of 0.736 (95% CI 0.602 – 0.870).

In this pilot evaluation of the VISA screening tool, sensitivity of 90.24% and specificity of 85.29% were found. The positive and negative predictive values were 93.67% and 78.36% respectively. These calculations are outlined in Table 1.

For peer review only - http://bmjopen.bmj.com/site/about/guidelines.xhtml

Table 1: Calculations of sensitivity, specificity and predictive values

Positive	
True positive, i.e. visual impairment present and referred	74
False negative, i.e. visual impairment present but not referred	8
Negative	
False positive, i.e. visual impairment not present but referred	5
True negative, i.e. visual impairment not present and not referred	29
Output	
Sensitivity (true positive/true positive + false negative)	90.24%<br>(95% CI: 81.68 – 95.69%)
Specificity (true negative/false positive + true negative)	85.29%<br>(95% CI: 68.94 – 95.05%)
Positive predictive value (true positive/false positive + true positive)	93.67%<br>(95% CI: 86.78 – 97.09%)
Negative predictive value (true negative/false negative + true negative)	78.38%<br>(95% CI: 64.91 – 87.66%)

Agreement was found for 103 participants (29 had no visual impairment, 74 required referral because of failed screening), outlined in Figure 1. The VISA screen produced eight false negative and five false positive results. Of the false negative results, three had ocular motility problems, three had reduced distance vision, one had reduced near vision and one did not have visual fields tested during screening. For false positive results, two with visual inattention, two with visual field loss and one with both visual inattention and visual field loss, were detected by screening and found not to be present by the vision assessment.

Test component agreement

The agreement for the individual components between the VISA screen and specialist vision assessments are outlined in Table 2. The highest levels of agreement were produced for distance visual acuity (0.785) and visual fields (0.741). The lowest levels of agreement were produced for ocular motility (0.120) and visual inattention (0.361). Low agreement for ocular motility related to high false negatives where 21 cases (3 with multiple conditions) were not detected - these comprised of: nine defects of vertical movement (including four age-related restrictions, one 4th cranial nerve palsy and one V-pattern), eight cases of nystagmus (including four end-point

For peer review only - http://bmjopen.bmj.com/site/about/guidelines.xhtml

nystagmus), five restrictions of horizontal eye movements and four cases of reduced convergence. The low agreement with visual inattention related to false positive referrals because of failure of the patient to complete this section due to impaired cognitive ability or fatigue – rather than true presence of visual inattention.

Table 2: Summary of agreement between the VISA screen and specialist vision assessment for referral to specialist eye services and individual components

Element of testing	Agreement	False negative	False positive	Kappa value (95% CI)
Referral	103	8	5	0.736 (0.602 – 0.870)
Near visual acuity	93	10	7	0.682 (0.543 – 0.820)
Distance visual acuity	94	8	3	0.785 (0.665 – 0.904)
Ocular alignment	112	4	0	0.585 (0.221 – 0.949)
Ocular motility	89	21	6	0.120 (-0.071 – 0.311)
Visual fields	94	3	8	0.741 (0.599 – 0.884)
Visual inattention	67	1	16	0.361 (0.144 – 0.578)

Naïve versus non-naïve screeners

The agreement on whether to make a referral to specialist eye services based on results of the VISA screening tool versus those from specialist vision assessment was stronger when made by a non-naïve screener (Table 3). A higher rate of false positive and false negatives were found when the screener was naïve to vision testing (eleven false referrals for naïve vs two for non-naïve screeners). The agreement on whether to make a referral to specialist eye services between the VISA screening tool and a specialist vision assessment had a Kappa value of 0.736 (95% CI 0.602 – 0.870).

When used by a naïve screener the VISA screen has a sensitivity of 82.93% and specificity of 80.95%. When used by non-naïve screeners the sensitivity the VISA screen has a sensitivity of 97.56% and specificity of 92.31%.

Table 3: Summary of agreement between the VISA screening tool and specialist vision assessment for referral to specialist eye services when used by a naïve versus non-naïve screener.

Screener	Agreement	False negative	False positive	Kappa value (95% CI)
Medical student	51	7	4	0.617 (0.415 – 0.820)

For peer review only - http://bmjopen.bmj.com/site/about/guidelines.xhtml

n=62				
Independent orthoptist<br>n=54	52	1	1	0.899 (0.761 – 1.000)

Process evaluation

Information from feedback sheets and detailed notes from interviews were compiled and grouped for type of feedback. Group themes included instruction feedback, section feedback and referral feedback.

Instruction feedback: Screeners asked for brief instruction reminders at the top of VISA screening assessments, for example, position test chart at 3 metres from the patient, cover each eye in turn, etc. This served to act as a quick reminder for the correct procedure for that particular section of the screening tool. Clarifications were requested for the main instruction training section such that potential ambiguity was removed.

Section feedback: In the first version, each screening section was coupled to the detailed assessment instructions. Screeners requested that all detailed instructions be merged into one training 'manual' section with the screening assessments separate. As screeners became more familiar with the tool, they used the VISA screens on their own and kept the detailed instructions elsewhere (mainly for reference) which meant there was less paperwork to be carried to the bedside assessment.

Referral feedback: Most feedback concerned patients who were borderline on whether to refer for specialist vision assessment or not. For example, where the patient had borderline visual acuity responses – perhaps because glasses were not available – but all other visual function assessments passed the VISA screen. In other cases, the patient lacked sufficient cognitive or communication abilities rendering some VISA screens 'unsure' or incomplete. Detailed referral guidelines were compiled to guide the referral process with minimum guidance being to repeat the VISA screen 1-2 days later for borderline cases. This aimed to reduce the levels of false referrals.

Discussion

In this study, we present the VISA screening tool which encompasses screening of key visual functions affected by stroke; namely central vision, peripheral visual field, eye position/movements and visual attention, alongside ocular history. Overall, referral had sensitivity and specificity of about 90% and 80% respectively, positive and negative predictive values of about 94% and 78% respectively, with agreement between VISA screening and comprehensive specialist assessment of above Kappa 0.7. Agreement was lowest for eye movement screening and visual inattention whereas all other individual sections showed agreement of above Kappa 0.5. Low agreement in

For peer review only - http://bmjopen.bmj.com/site/about/guidelines.xhtml

these sections related to high false positive referrals where VISA screen indicated a fail for ocular motility or visual inattention. The specialist vision assessment detected ocular motility changes which were classed as 'normal' physiological eye movement patterns such as V pattern and end-point nystagmus, and which alone would not have required referral. The detection of these physiological eye movement patterns was regarded as a positive finding within the eye movement section indicating that the ocular motility section had proved to be sensitive to these less obvious eye movement problems. However, this section requires close monitoring in further studies to refine related training and referral guidelines. False positive referrals for visual inattention occurred where the patient failed to complete the section because of fatigue or cognitive impairment. The incomplete results were interpreted as borderline fail by screeners. Visual inattention was the last section to be completed in the VISA screen so, as a result, were likely to be most susceptible to the effects of fatigue and impaired cognition. Guidance on completing the VISA screen was therefore amended such that the more interactive components of the VISA screen (i.e. visual field and visual inattention sections) were advised to be completed first in cases where cognition or fatigue could impact on screen complete; plus a repeat second screen was advised where indicated.

Process evaluation aided further refinement of the VISA screening tool and, in particular, training elements and referral guidance to add quick tips and reminders, and to remove ambiguity. Vision screening of stroke survivors by orthoptists using validated assessments has been shown to provide accurate identification of visual impairment and is easily undertaken on the stroke unit with further follow-up arranged in eye clinics as required (13). Such Orthoptic input has been reported to help prevent misdiagnosis, provide quick access to treatment of visual problems and improve response to general rehabilitation (4, 14). Orthoptists are a member of the core acute stroke MDT (10). Despite consistent findings that inclusion of vision services within the MDT is highly beneficial, such visual assessment is not common and services are inconsistent throughout the UK. One survey showed that 45% of stroke services provided no formal vision assessment for stroke patients (15). A further survey of practice identified that only 7% of stroke units had a policy relating to vision assessment and management (16). Both surveys showed lack of standardisation for vision assessment and treatment for stroke survivors. The National Stroke Strategy argues that vision and visual perceptual difficulties are components requiring multi-faceted stroke specific rehabilitation and support (17). The Royal College of Physicians recommend that every patient with stroke should have a practical assessment of vision and examination of the visual field (10).

Problems exist with referral accuracy from the MDT where there is suspected visual difficulty. It is reported that where referral by the MDT was based on the identification of ocular signs *only*, there

For peer review only - http://bmjopen.bmj.com/site/about/guidelines.xhtml

was reduced sensitivity (42%) and specificity (52%) (3). Referral accuracy improved when visual symptoms were taken into account. Concerns were raised regarding potential failure to refer those patients unable to report their visual symptoms due to communication and cognitive deficits (3). Inconsistencies between identification of ocular signs on assessment by the MDT and final ocular diagnosis have also been documented in an audit of stroke referrals for vision assessment (18). Fifty-six percent of visual diagnoses made prior to formal eye assessment were incorrect with amended diagnoses being made following visual assessment by the orthoptic/ophthalmic team (18). Our VISA screen at this early pilot stage appears to increase the accuracy of screening by increasing the ability to detect ocular signs separate to reporting of vision symptoms.

In each of the above studies, the MDT used a screening form on which they specified whether they noted any obvious visual signs such as nystagmus, strabismus or ptosis and whether the patient complained of visual symptoms such as double vision or reading difficulty. They did not, however, undertake any measurement of visual function. A further study evaluated Cardiff cards as a screening measure to identify low levels of vision (19). A comparative study of qualitative methods of visual field assessment reported the difficulty in screening for visual field impairment in acute stages of stroke follow-up (20). However, the authors recognised that confrontation is widely regarded as the most viable screening option for bedside visual field assessment (19). Visual inattention is the most common visual perceptual disorder and there are various screening assessments in use for its detection but which do not extend to other facets of visual impairment (21). In each of these studies, individual assessments of one aspect of visual function are considered. However, an overall visual screening assessment for stroke survivors is currently not available for use by MDTs in the absence of assessment by eye care professionals (21).

Limitations: The VISA screening tool was used by a combination of medial students and orthoptists whilst specialist vision assessment was provided by a team of orthoptists and ophthalmologists. Arguably, results wold be more meaningful if all VISA screens were completed by staff naïve to any vision assessment. Because this was a pilot validation study, we chose to include VISA screens from both medical students with no vision assessment experience, and orthoptists who were experienced in vision assessment. Medical students represented completely naïve individuals in conducting vision screening assessments. However, orthoptists were chosen as screeners in this pilot stage of validation to serve as a quality check of the screening tool's ability to accurate assess various aspects of visual impairment. Our process evaluation for acceptability of the VISA screen involved feedback and interviewers with screeners only. We acknowledge this limitation and an important next step is to obtain views of stroke survivors on the acceptability of the VISA screen and its perceived value to them. A further limitation is that the VISA screen was not timed consistently for duration.

For peer review only - http://bmjopen.bmj.com/site/about/guidelines.xhtml

Completion of the VISA screen was approximately 10 minutes in the small number that could be assessed but this cannot be taken as a representative screen duration. The screening duration is an important consideration when adding to busy acute stroke services and will be captured fully in the next stage of validation.

Our next stage of development is a full clinical validation of the VISA tool where all screening assessments are completed by naïve screeners versus reference comprehensive vision assessment.

Conclusions

This early validation of the VISA screening tool shows promise in improving detection accuracy for clinicians involved in stroke care who are not specialists in vision problems and lack formal eye training, with potential to lead to more prompt referral with fewer false positives and negatives. Clinicians reported acceptability of the VISA screening tool for is use in screening for presence of vision problems in stroke survivors. Referral sensitivity of 90% and specificity of 80% were found for the VISA screening with strong inter-rater agreement for referral between VISA screening and specialist vision assessments.

The benefits are that the VISA screening tool may support increased speed of access to appropriate treatment of visual impairment and potential to preserve and make best use of remaining visual function for patients. Identification of visual impairment and implementation of early interventions and compensatory options has impact to overall rehabilitation, quality of life and activities of daily living with potential cost savings to the NHS by enhancing rehabilitation and supporting early discharge. Establishment of an effective vision screening tool is likely to be highly transferable to other vulnerable groups in other hospital in-patient areas, residential care settings or community multidisciplinary team assessments.

Funding statement

This study received no specific grant from any funding agency in the public, commercial or not-for-profit sectors'. FR and CH are part funded by National Institute for Health Research fellowship awards.

Competing interests

The authors have no competing interests to declare.

Acknowledgements

For peer review only - http://bmjopen.bmj.com/site/about/guidelines.xhtml

The authors would like to express thanks to the staff and patients at Salford Royal Hospital and the Walton Centre.

Author contributions

All authors (FR, LH, KH and CH) were involved in study set-up and data collection. LH and FR carried out the data analysis and wrote the initial manuscript draft. The manuscript was critically reviewed by KH and CH. All authors read and approved the final manuscript.

Data sharing

No additional data available

References

1. Hepworth LR, Rowe FJ, Walker MF, Rockliffe J, Noonan C, Howard C, Currie J. Post-stroke visual impairment: a systematic literature review of types and recovery of visual conditions. Ophthalmology Research. 2016; 5(1). DOI: 10.9734/OR/2016/21767.

2. Rowe F, VIS writing group. Vision In Stroke cohort: profile overview of visual impairment. Brain and Behavior. 2017. DOI: 10.1002/brb3.771.

3. Rowe F, Wright D, Brand D, Jackson C, Price A, Walker L, Harrison S, Eccleston C, Maan T, Scott C, Vogwell L, Peel S, Robson L, Akerman N, Dodridge C, Howard C, Shipman T, Sperring U, Yarde S, MacDiarmid S, Freeman C. Reading difficulty after stroke: ocular and non ocular causes. International Journal of Stroke. 2011; 6(5): 404-411.

4. Jones SA, Shinton RA. Improving outcome in stroke patients with visual problems. Age and Ageing. 2006; 35(6): 560-565.

5. Hanna KL, Hepworth LR, Rowe FJ. The treatment methods for post-stroke visual impairment: a systematic review. Brain and Behavior. 2017; 7(5). DOI: 10.1002/brb3.682.

6. Tsai S-Y, Cheng C-Y, Hsu W-M, Su T-PT, Liu J-H, Chou P. Association between visual impairment and depression in the elderly. Journal of Formosan Medical Association. 2003; 102(2): 86-90.

7. The James Lind Alliance. Top 10s of priorities for research. 2017 [Available from: http://www.jla.nihr.ac.uk/top-10-priorities/ [Accessed: 13 June 2017].

8. Rowe F. Impact of Visual Impairment after Stroke: the IVIS study. 2014 [Available from: www.ivis.org.uk/ [Accessed:

For peer review only - http://bmjopen.bmj.com/site/about/guidelines.xhtml

9. Aho K, Harmsen P, Hatano S, Marquardsen J, Smirnov VE, Strasser T. Cerebrovasular disease in the community: results of a WHO Collaborative Study. Bulletin of the World Health Organisation. 1980; 58(1): 113-130.

10. Intercollegiate Stroke Working Party. National clinical guideline for stroke. 5th ed. London: Royal College of Physicians; 2016.

11. British and Irish Orthoptic Society. Compentency standards and professional practice guidelines: extended practioner role Section 2: Stroke assessment and rehabilitation. London: British and Irish Orthoptic Society,; 2011.

12. Bland JM, Altman DG. Statistical methods for assessing agreement between two methods of clinical measurement. Lancet. 1986; 327(8476): 307-310.

13. MacIntosh C. Stroke re-visited: visual problems following stroke and their effect on rehabilitation. British Orthoptic Journal. 2003; 60: 10-14.

14. Rowe F, Brand D, Jackson CA, Price A, Walker L, Harrison S, Eccleston C, Scott C, Akerman N, Dodridge C, Howard C, Shipman T, Sperring U, MacDiarmid S, Freeman C. Visual impairment following stroke: do stroke patients require vision assessment? Age and Ageing. 2009; 38(2): 188-193.

15. Rowe FJ. Who sees visual impairment following stroke? Strabismus. 2010; 18(2): 37-40.

16. Pollock A, Brady M, Hazelton C. Management of visual problems after stroke: a survey of current practice in Scotland. International Journal of Stroke. 2009; 4(Suppl 2): 44.

17. Department of Health. National Stroke Strategy. London: DH Publications; 2007.

18. Brand D. 2009.

19. Johansen A, White S, Waraisch P. Screening for visual impairment in older people: validation of the Cardiff Acuity Test. 36. 2003; 3(289-293).

20. Cassidy TP, Bruce DW, Gray CS. Visual field loss after stroke: confrontation and perimetry in the assessment of recovery. Journal of Stroke and Cerebrovascular Diseases. 2001; 10(3): 113-117.

21. Hanna KL, Hepworth LR, Rowe FJ. Screening methods for post-stroke visual impairment: a systematic review. Disability and Rehabilitation. 2017; 39(25): 2531-2543.

Figure 1
Flow diagram of participant outcome for screening and full assessment

For peer review only - http://bmjopen.bmj.com/site/about/guidelines.xhtml

Figure 1: Flow diagram of participant outcome for screening and full assessment

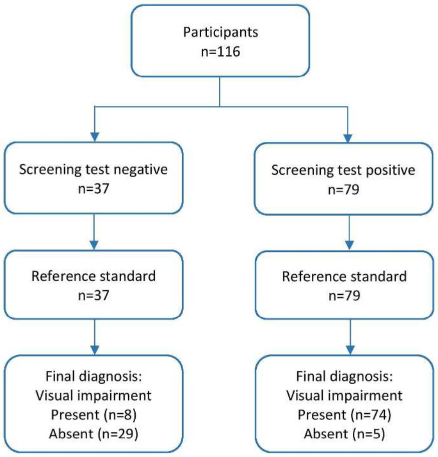

Flow diagram of participant outcome for screening and full assessment

210x297mm (300 x 300 DPI)

Section & Topic	No	Item	Reported on page #
**TITLE OR ABSTRACT**			
	**1**	Identification as a study of diagnostic accuracy using at least one measure of accuracy (such as sensitivity, specificity, predictive values, or AUC)	#2
**ABSTRACT**			
	**2**	Structured summary of study design, methods, results, and conclusions (for specific guidance, see STARD for Abstracts)	#2
**INTRODUCTION**			
	**3**	Scientific and clinical background, including the intended use and clinical role of the index test	#3
	**4**	Study objectives and hypotheses	#3
**METHODS**			
Study design	**5**	Whether data collection was planned before the index test and reference standard were performed (prospective study) or after (retrospective study)	#4
Participants	**6**	Eligibility criteria	#4
	**7**	On what basis potentially eligible participants were identified (such as symptoms, results from previous tests, inclusion in registry)	#4
	**8**	Where and when potentially eligible participants were identified (setting, location and dates)	#4
	**9**	Whether participants formed a consecutive, random or convenience series	#4
Test methods	**10a**	Index test, in sufficient detail to allow replication	#4
	**10b**	Reference standard, in sufficient detail to allow replication	#4
	**11**	Rationale for choosing the reference standard (if alternatives exist)	#4
	**12a**	Definition of and rationale for test positivity cut-offs or result categories of the index test, distinguishing pre-specified from exploratory	#5
	**12b**	Definition of and rationale for test positivity cut-offs or result categories of the reference standard, distinguishing pre-specified from exploratory	#5
	**13a**	Whether clinical information and reference standard results were available to the performers/readers of the index test	#5
	**13b**	Whether clinical information and index test results were available to the assessors of the reference standard	#5
Analysis	**14**	Methods for estimating or comparing measures of diagnostic accuracy	#5
	**15**	How indeterminate index test or reference standard results were handled	#5
	**16**	How missing data on the index test and reference standard were handled	#6
	**17**	Any analyses of variability in diagnostic accuracy, distinguishing pre-specified from exploratory	#7
	**18**	Intended sample size and how it was determined	#5
**RESULTS**			
Participants	**19**	Flow of participants, using a diagram	Figure 1
	**20**	Baseline demographic and clinical characteristics of participants	#5
	**21a**	Distribution of severity of disease in those with the target condition	
	**21b**	Distribution of alternative diagnoses in those without the target condition	
	**22**	Time interval and any clinical interventions between index test and reference standard	#4
Test results	**23**	Cross tabulation of the index test results (or their distribution) by the results of the reference standard	#6-7
	**24**	Estimates of diagnostic accuracy and their precision (such as 95% confidence intervals)	#6
	**25**	Any adverse events from performing the index test or the reference standard	
**DISCUSSION**			
	**26**	Study limitations, including sources of potential bias, statistical uncertainty, and generalisability	#11
	**27**	Implications for practice, including the intended use and clinical role of the index test	#11
**OTHER INFORMATION**			
	**28**	Registration number and name of registry	
	**29**	Where the full study protocol can be accessed	
	**30**	Sources of funding and other support; role of funders	#12

For peer review only

STARD 2015

AIM

STARD stands for "Standards for Reporting Diagnostic accuracy studies". This list of items was developed to contribute to the completeness and transparency of reporting of diagnostic accuracy studies. Authors can use the list to write informative study reports. Editors and peer-reviewers can use it to evaluate whether the information has been included in manuscripts submitted for publication.

EXPLANATION

A **diagnostic accuracy study** evaluates the ability of one or more medical tests to correctly classify study participants as having a **target condition.** This can be a disease, a disease stage, response or benefit from therapy, or an event or condition in the future. A medical test can be an imaging procedure, a laboratory test, elements from history and physical examination, a combination of these, or any other method for collecting information about the current health status of a patient.

The test whose accuracy is evaluated is called **index test.** A study can evaluate the accuracy of one or more index tests. Evaluating the ability of a medical test to correctly classify patients is typically done by comparing the distribution of the index test results with those of the **reference standard**. The reference standard is the best available method for establishing the presence or absence of the target condition. An accuracy study can rely on one or more reference standards.

If test results are categorized as either positive or negative, the cross tabulation of the index test results against those of the reference standard can be used to estimate the **sensitivity** of the index test (the proportion of participants *with* the target condition who have a positive index test), and its **specificity** (the proportion *without* the target condition who have a negative index test). From this cross tabulation (sometimes referred to as the contingency or "2x2" table), several other accuracy statistics can be estimated, such as the positive and negative **predictive values** of the test. Confidence intervals around estimates of accuracy can then be calculated to quantify the statistical **precision** of the measurements.

If the index test results can take more than two values, categorization of test results as positive or negative requires a **test positivity cut-off**. When multiple such cut-offs can be defined, authors can report a receiver operating characteristic (ROC) curve which graphically represents the combination of sensitivity and specificity for each possible test positivity cut-off. The **area under the ROC curve** informs in a single numerical value about the overall diagnostic accuracy of the index test.

The **intended use** of a medical test can be diagnosis, screening, staging, monitoring, surveillance, prediction or prognosis. The **clinical role** of a test explains its position relative to existing tests in the clinical pathway. A replacement test, for example, replaces an existing test. A triage test is used before an existing test; an add-on test is used after an existing test.

Besides diagnostic accuracy, several other outcomes and statistics may be relevant in the evaluation of medical tests. Medical tests can also be used to classify patients for purposes other than diagnosis, such as staging or prognosis. The STARD list was not explicitly developed for these other outcomes, statistics, and study types, although most STARD items would still apply.

DEVELOPMENT

This STARD list was released in 2015. The 30 items were identified by an international expert group of methodologists, researchers, and editors. The guiding principle in the development of STARD was to select items that, when reported, would help readers to judge the potential for bias in the study, to appraise the applicability of the study findings and the validity of conclusions and recommendations. The list represents an update of the first version, which was published in 2003.

More information can be found on http://www.equator-network.org/reporting-guidelines/stard.

cc BY SA

For peer review only - http://bmjopen.bmj.com/site/about/guidelines.xhtml
